# Beneficial switch from aflibercept to ranibizumab for the treatment of refractory neovascular age-related macular degeneration

**DOI:** 10.1007/s00417-020-04730-8

**Published:** 2020-05-12

**Authors:** Liza-Marie Marquis, Irmela Mantel

**Affiliations:** Department of Ophthalmology, University of Lausanne, Jules Gonin Eye Hospital, Foundation Asile des Aveugles, 15 Avenue de France, CP 5143, CH-1000 Lausanne, Switzerland

**Keywords:** Aflibercept, Ranibizumab, Switch, Refractory, Neovascular age-related degeneration, Anti-VEGF

## Abstract

**Purpose:**

The aim of this study was to evaluate the effects of switching to ranibizumab in patients with neovascular age-related macular degeneration (nAMD) refractory to aflibercept treatment and to identify predictive factors for switch response.

**Methods:**

A retrospective chart review was conducted including 32 eyes from 26 patients with refractory nAMD, who switched from monthly intravitreal aflibercept treatment (≥ 6 months) to ranibizumab. Outcome measures included changes in visual acuity (VA), intraretinal fluid (IRF), subretinal fluid (SRF), pigment epithelial detachment (PED), and central retinal thickness (CRT), evaluated at 6 months before switch (T1), at the time of switch (T2), and 3 months post-switch (T3).

**Results:**

There was an increase in CRT from T1 to T2, which decreased after switch from T2 to T3. Regression analysis of the changes per month observed between time points showed significant differences in PED height (*p =* 0.02), SRF (*p =* 0.01), and neuroretinal thickness as a measure for IRF (*p =* 0.03). No significant change was found for VA. Predictive factors for better switch response included an exacerbation between T1 and T2, thicker measurements at T2, male sex, shorter treatment duration before switch, and fewer preceding injections. No association with preceding switch was found.

**Conclusion:**

Patients with nAMD refractory to aflibercept benefit from switching to ranibizumab, particularly those whose condition worsened prior to the switch. This may be explained by drug tolerance to aflibercept. Our findings may facilitate making appropriate treatment decisions, potentially improving patient outcomes.

## Introduction

Age-related macular degeneration (AMD) is a frequent retinal disorder in the elderly, which can lead to the loss of central vision and legal blindness [1, 2]. Neovascular AMD (nAMD) is a late form of AMD characterized by choroidal neovascularization leading to macular exudation and ultimately fibrosis with severe vision loss. Intravitreal anti-vascular endothelial growth factor (anti-VEGF) agents have been found to improve visual acuity (VA) outcomes in nAMD [3, 4]. Two anti-VEGF drugs are most widely used based on their effectiveness and FDA approval: ranibizumab, which was first commercialized in 2006, and aflibercept, which has been available since 2011. These two drugs differ from each other in terms of their structure and pharmacological action. Ranibizumab is a humanized monoclonal antibody antigen-binding fragment that blocks VEGF-A, whereas aflibercept is a soluble recombinant fusion protein composed of VEGF receptors 1 and 2. Consequently, in contrast to ranibizumab, aflibercept inhibits not only VEGF-A but also VEGF-B and placental growth factor, all of which are thought to be involved in nAMD pathology [5]. Aflibercept has a higher affinity and a longer activity than ranibizumab, at least in theory [5–7]. However, both drugs seem to have similar visual benefits when applied in nAMD treatment [3].

Retreatment need of nAMD patients, which is necessary in order to control for exudative recurrences, is highly variable between affected individuals. While the mean need for retreatment is approximately every 2 months, some patients present with intraretinal fluid (IRF) and/or subretinal fluid (SRF) despite the maximal dosage of monthly retreatment [3]. These patients are commonly referred to as refractory to the respective drug, although this does not imply an absence of but rather an insufficiency of treatment effect [8]. Drug tolerance, among others, has been suggested as a possible reason for refractoriness [9]. Improvement of the structural response has been extensively reported after drug switch from ranibizumab to aflibercept [10–17]. Whether the effectiveness of this drug switch is attributable to a circumvention of drug tolerance or the different pharmacologic behaviors of the drugs has been speculated. However, there is little evidence about the effect of switching from aflibercept to ranibizumab [18–20]. Such evidence could help to elucidate the role of drug tolerance versus pharmacological differences. In addition, it would be helpful for making appropriate and individual clinical decisions.

The aim of this study was to evaluate the effects of switching from aflibercept to ranibizumab in patients with refractory nAMD and to identify potential predictive factors.

## Methods

This study was a retrospective chart review performed at the medical retina unit of the Jules Gonin University Eye Hospital in Lausanne, Switzerland. The study was approved by the Swiss Federal Department of Health for retrospective data analysis and was performed in accordance with the ethical standards of the Declaration of Helsinki. This study did not require informed consent.

A consecutive series of patients with nAMD, refractory to monthly anti-VEGF therapy using aflibercept, and having switched to ranibizumab were identified from the institutional database. Refractoriness was defined as evidence of IRF or SRF on spectral domain optical coherence tomography (SD-OCT) at each monthly visit for 6 months or more, despite monthly aflibercept injection. Exclusion criteria were the presence of any retinal pathology other than nAMD, poor OCT image quality, and interruption of treatment.

The routine treatment protocol comprised a series of 3 monthly injections, which, if fluid was still present, was repeated. If nAMD showed refractoriness despite treatment ongoing for at least 12 months, anti-VEGF switch was considered. The choice of the first anti-VEGF used was not guided by any clinical ocular parameter. Clinicians tended to use first ranibizumab due to its ease of preloaded syringes. However, during the year 2013, we recruited treatment naïve patients for a 2-year study using aflibercept in 112 eyes [21]. In case of refractoriness at the end of this study, they were typically switched to ranibizumab. After switching to ranibizumab, patients went through another series of 3 monthly injections. If a dry macula was achieved at the end of this regimen, injection intervals were extended thereafter from 1 to 1.5 months according to the observe and plan regiment [21–23].

For each included patient and eye, the following data were collected: best available VA, routinely measured on the Early Treatment Diabetic Retinopathy Study (ETDRS) chart; presence or absence of IRF and SRF on SD-OCT; the respective maximal thickness on the vertical A-scan; presence or absence of pigment epithelial detachment (PED) and its maximum height; and the central retinal thickness (CRT) as measured by the integrated software of the OCT Spectralis device (Heidelberg Engineering, Dossenheim, Germany). Each of the above parameters was evaluated at three different time points. The T1 time corresponded to 6 months before switch. The T2 time corresponded to the time of the drug switch, and T3 time was 3 months after switch. Further, collected data included age, sex, eye, date of initiation of aflibercept treatment, number of injections received in the study eye, and previous anti-VEGF switches.

Outcome measures were changes in VA, CRT, dryness of the macula, SRF height, and PED height. Variables analyzed for their impact on these outcome measures included the corresponding change during the 6 months preceding the switch, the absolute value at T2, age, sex, number of previous injections, previous switch, and the duration of aflibercept treatment.

The statistical analysis included descriptive statistics and a paired analysis of continuous variables to identify significant changes over time. An association analysis was performed to identify factors associated with the structural outcome measures after switch, using Pearson’s correlation analysis for continuous variables and ANOVA for categorical variables. The JMP statistical program for Windows (version 8.0.1, SAS institute Inc., Cary, NC) was used. Data are expressed as mean ± standard deviation (SD). *P* < 0.05 was considered statistically significant.

## Results

The inclusion criteria were met by 32 eyes (19 right and 13 left eyes) from 26 patients with a mean age of 75.5 ± 7.8 years, 18 of which were female (69%). The proportion of occult subtype was 30 out of 32 eyes (93.8%). Classic neovascularization was present in one and angiomatous proliferation in one eye. Patients received an average of 34.5 ± 13.4 intravitreal injections of anti-VEGF over 3.5 ± 1.4 years. Sixteen eyes (50%) had already been previously switched (from ranibizumab to aflibercept). The preceding switch (from ranibizumab to aflibercept) in these 50% of cases had been performed with a mean of 17.4 months (SD 9.8, range 7.2 to 39.7 months) before the investigated switch. The number of injections (aflibercept) given between the first and the second switch was 16.4 (SD 8.7, range 7 to 39).

### Comparison between time points

Table 1 summarizes the means and SDs of VA, CRT, PED, and SRF with corresponding *p* values, and the number of eyes with presence or absence of IRF, at the different time points (T1, T2, T3). While CRT, SRF, and PED increased from T1 to T2, the opposite was found between T2 and T3. These differences only partially reached statistical significance (Table 1). However, the comparison between T1 and T3 was not significantly different. VA decreased slightly, both from T1 to T2 and from T2 to T3. The VA change reached significance when comparing T1 with T3.

**Table 1:** Visual and anatomical measurements at the different time points

Outcome measure	T1	T2		T3		
	Mean ± SD	Mean ± SD	*p* value compared with T1	Mean ± SD	*p* value compared with T2	*p* value compared with T1
VA in ETDRS letters	74.9 ± 12.8	72.3 ± 14.2	0.20	72.0 ± 13.5	0.85	0.07
CRT in μm thickness	379 ± 126	404 ± 109	0.13	393 ± 136	0.48	0.12
PED in μm height	234 ± 113	243 ± 111	0.16	226 ± 105	0.03	0.20
SRF in μm height	40 ± 44	78 ± 78	0.03	55 ± 77	0.06	0.71
	T1 Number of eyes (%)	T2 Number of eyes (%)		T3 Number of eyes (%)		
Presence of IRF	17 (53%)	18 (56%)		18 (56%)		
Presence of SRF	18 (56%)	24 (75%)		19 (59%)		

Statistical test, paired *t* test

*VA*, visual acuity; *ETDRS*, Early Treatment Diabetic Retinopathy Study; *CRT*, central retinal thickness; *PED*, pigment epithelial detachment; *SRF*, subretinal fluid; *SD*, standard deviation; *T1*, 6 months before the switch; *T2*, switch time point; *T3*, 3 months post-switch

The presence of IRF changed very little between time points (only 1 case with new IRF from T1 to T2, Table 1), while SRF showed more fluctuation with a clear increase from T1 (18 eyes) to T2 (24 eyes) and a decrease from T2 to T3 (19 eyes). Complete dryness, i.e., the absence of IRF and SRF, was achieved in 4 eyes (13%) at T3.

### Regression analysis of the changes before and after switch (slope)

As there was a clear effect switch on the evolution of exudative signs before and after drug switch, we compared not only the individual time points but also the individual regression coefficients (slopes) before and after switch. Paired analysis revealed statistically significant changes: The slope (regression coefficient) of PED changed significantly from increase (from T1 to T2) to a decrease (T2 to T3) (*p* = 0.02), as did the slope of SRF (*p =* 0.01) and neuroretinal thickness with IRF (*p =* 0.03). No significant changes in regression coefficients were found for CRT or VA.

### Factor analysis related to the changes after switch

A number of candidate factors that were identified to possibly be associated with the degree of change following drug switch were analyzed using univariate and multivariate analysis. The results are summarized in Table 2.

**Table 2:** Univariate and multivariate factor analysis related to the changes after switch

	CRT			IRF			SRF			PED		
	Univariate analysis		Multivariate analysis	Univariate analysis		Multivariate analysis	Univariate analysis		Multivariate analysis	Univariate analysis		Multivariate analysis
	*R*^2^	*p* value	*p* value	*R*^2^	*p* value	*p* value	*R*^2^	*p* value	*p* value	*R*^2^	*p* value	*p* value
T1-T2	− 0.69	< 0.0001	0.001	− 0.39	0.03	NS	− 0.54	0.001	0.0001	− 0.63	0.0001	0.0007
Absolute value at T2	− 0.15	0.36		− 0.44	0.01	NS	− 0.37	0.04	NS	− 0.31	0.08	NS
Age	− 0.24	0.14	NS	0.03	0.86		0.12	0.50		0.00	0.99	
Sex		0.17	NS		0.07	NS		0.52			0.01	0.04
Injections number	0.15	0.35		0.09	0.62		0.52	0.002	NS	0.06	0.76	
Years before switch	0.30	0.07	0.03	0.01	0.96		0.62	0.0002	0.0001	− 0.05	0.79	
Preceding switch		0.62			0.72			0.10	NS		0.87	

Statistical tests, Pearson correlation analysis for continuous variables and ANOVA test for categorical variables

*CRT*, central retinal thickness; *IRF*, intraretinal fluid; *SRF*, subretinal fluid; *PED*, pigment epithelial detachment; *R*^2^, correlation coefficient; *NS*, not significant

We found a significant association for all 4 outcome measures, i.e., CRT, IRF, SRF, and PED, between the changes observed before (T1 to T2) and after the switch (T2 to T3). This finding was confirmed in multivariate analysis for the outcomes of CRT, SRF, and PED, indicating that a greater increase prior to the switch predicted a greater decrease thereafter. Similarly, an association was seen for the changes in IRF and SRF after switch with the corresponding absolute thicknesses at T2, suggesting that the more pathological fluid was present, the better the eye responded to the switch in drugs. Furthermore, the PED response post-switch was associated with sex, in that male patients showed a stronger response than female patients did. A shorter pre-switch treatment period was associated with a better response to switch in SRF and CRT, which was confirmed by multivariate analysis.

However, other candidate factors, such as age, the number of injections received prior to switch, or having changed anti-VEGF drugs before, were not confirmed as predictors for the switch response from aflibercept to ranibizumab, except for the change in SRF, which correlated with the number of preceding injections.

## Discussion

In this retrospective study, we observed a promising response to ranibizumab treatment in the eyes with nAMD that had been refractory to monthly aflibercept. However, this response was found to be dependent on several specific factors, with a particularly strong impact of the degree of change before switch.

Similar to what has previously been reported for switching from ranibizumab to aflibercept [10–17], we found that the inverse switch can be effective in the treatment of nAMD as well. Therefore, at least part of the effect of switch may not be attributable to differences in drugs but rather a phenomenon such as drug tolerance [8, 9, 24]. Tolerance occurs when the response to a specific drug (aflibercept in this study) decreases after repeated intravitreal injections, possibly because of compensatory mechanisms. Such changes could include an increased expression of VEGF or VEGF receptors, secretion of other growth factors, interference of specific antibodies, or changes in signal transduction [25]. Typically, it becomes necessary to increase the anti-VEGF dosage or decrease the treatment interval to maintain the same effect as achieved at the beginning of the treatment [8]. The alternative explanation by tachyphylaxis appears not applicable in nAMD, as it would correspond to a rapid decrease in treatment effectiveness, which cannot be improved by drug dosage, but only by temporarily stopping treatment or increasing the interval between the intravitreal injections. This mechanism could typically occur in drugs releasing neurotransmitters [8].

In the current study, VA did not improve following the switch to ranibizumab, a finding consistent with many other studies on switching anti-VEGF drugs that have shown a beneficial response only in morphological parameters [10–12, 14, 16, 17, 20]. However, a few other groups did observe an improvement of VA after switching drugs [13, 15, 18]. Although the ultimate goal of clinical eye research remains visual function, improving structural aspects of the retina remains useful even in the absence of immediate effects on vision. Indeed, with prolonged duration of abnormal structural changes, functional limitations tend to become increasingly irreversible. Therefore, the attempt to achieve a retinal status closest possible to normal anatomy appears justified even without immediate visual effects. The absence of instantaneous changes in best corrected VA despite an observation of molecular and structural changes could be related to previous irreversible retinal damage [26]. No ocular adverse events, which could have distorted the functional results, have been observed in this study.

We found that switching from aflibercept to ranibizumab led to beneficial short-term results regarding retinal structure. Previous reports about switchback from aflibercept to ranibizumab have found similar results [18–20]. Compared with the monthly change before the switch, PED, IRF, and SRF showed significantly different, favorable changes post-switch. 

Novel findings of our study include the factors predicting the observed post-switch changes, which could be of great importance in clinical practice. The pre-switch structural changes under aflibercept negatively correlated with the post-switch response in morphological improvements to ranibizumab. This was true for all structural outcomes, i.e., CRT, IRF, SRF, and PED, and was consistent with our previous study, in which we examined the effects of a switch from ranibizumab to aflibercept for the treatment of nAMD [9]. Thus, evaluation of the pre-switch refractory treatment period, independent of which anti-VEGF drug is used, may be useful to identify which eyes are likely to benefit from a switch to another anti-VEGF agent. Furthermore, the absolute values of IRF and SRF at the time point of switch predicted the degree of response to switching to ranibizumab.

An additional predictive factor we identified was the duration of the aflibercept treatment before the switch to ranibizumab. It was an independent predictor for SRF and CRT, whereby the shorter the eyes had been treated with aflibercept, the better was their response to the new drug. While the reasons behind this observation are not clear, it could be speculated that chronicity of treatment reduces the structural reactivity, perhaps due to protein-rich fluids. Therefore, a new therapeutic strategy may involve brief treatment periods with a successive switch to another, related drug.

Surprisingly, also sex was found to be a predictive factor, whereby being male predicted the post-switch change, even though this was restricted to PED. To the best of our knowledge, no previous studies have identified sex as a factor influencing the response to the switch from one anti-VEGF drug to another. It is possible that the likelihood for the development of a tolerance toward aflibercept is related to sex, perhaps due to the anti-placental growth factor component of the drug, whereby women might show less immunogenicity to this component.

Limitations of the present study are the retrospective nature of its design and the short follow-up period. A longer observation would be necessary in order to evaluate potential long-term functional effects. The high percentage of occult neovascularization may confine the conclusions mainly to this subtype. In addition, the sample size was small, and CRT measures were not manually corrected for segmentation errors.

In conclusion, the current study revealed encouraging results regarding structural aspects of refractory nAMD eyes following the switch from aflibercept to ranibizumab. Several factors were identified to predict which eyes benefit most from such a treatment change. In addition to retinal thickness, a particularly strong predictive factor was the short history of worsening of exudation just before switching, which increased the chances of a beneficial outcome. Further studies will be needed to confirm our results, ideally with a prospective design and a larger cohort.
